# Quantitative Evaluation of the Pore and Window Sizes of Tissue Engineering Scaffolds on Scanning Electron Microscope Images Using Deep Learning

**DOI:** 10.1021/acsomega.4c01234

**Published:** 2024-05-10

**Authors:** Ilayda Karaca, Betül Aldemir Dikici

**Affiliations:** Department of Bioengineering, Izmir Institute of Technology, Urla, Izmir 35433, Turkey

## Abstract

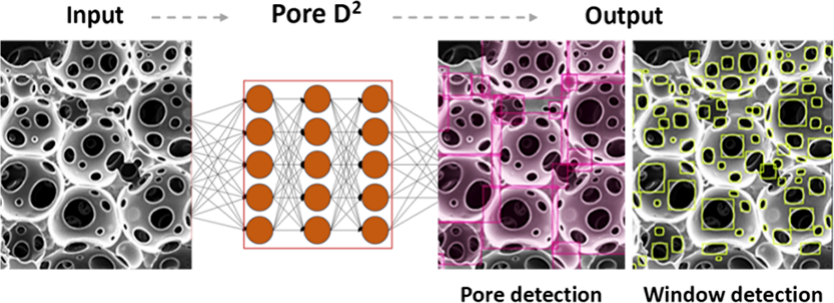

The morphological characteristics of tissue engineering scaffolds, such as pore and window diameters, are crucial, as they directly impact cell-material interactions, attachment, spreading, infiltration of the cells, degradation rate and the mechanical properties of the scaffolds. Scanning electron microscopy (SEM) is one of the most commonly used techniques for characterizing the microarchitecture of tissue engineering scaffolds due to its advantages, such as being easily accessible and having a short examination time. However, SEM images provide qualitative data that need to be manually measured using software such as ImageJ to quantify the morphological features of the scaffolds. As it is not practical to measure each pore/window in the SEM images as it requires extensive time and effort, only the number of pores/windows is measured and assumed to represent the whole sample, which may cause user bias. Additionally, depending on the number of samples and groups, a study may require measuring thousands of samples and the human error rate may increase. To overcome such problems, in this study, a deep learning model (Pore D^2^) was developed to quantify the morphological features (such as the pore size and window size) of the open-porous scaffolds automatically for the first time. The developed algorithm was tested on emulsion-templated scaffolds fabricated under different fabrication conditions, such as changing mixing speed, temperature, and surfactant concentration, which resulted in scaffolds with various morphologies. Along with the developed model, blind manual measurements were taken, and the results showed that the developed tool is capable of quantifying pore and window sizes with a high accuracy. Quantifying the morphological features of scaffolds fabricated under different circumstances and controlling these features enable us to engineer tissue engineering scaffolds precisely for specific applications. Pore D^2^, an open-source software, is available for everyone at the following link: https://github.com/ilaydakaraca/PoreD2.

## 1 Introduction

Tissue engineering is an interdisciplinary field that has emerged to develop biological substitutes to repair tissues or improve their function using the principles of engineering and life sciences.^1^ To regenerate damaged areas, tissue engineering involves using biodegradable porous matrices (also known as tissue engineering scaffolds) made from biomaterials that mimic the biochemical and structural characteristics of natural tissues.

**Figure 1:**
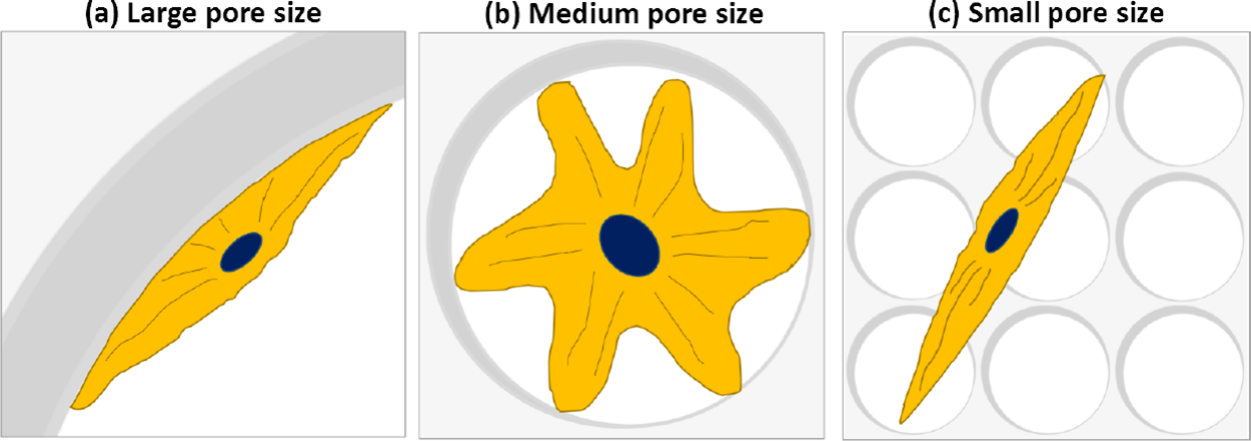
Attachment, spreading, and morphology of a cell on the scaffold with (a) large, (b) medium, and (c) small pore diameter.

Features associated with the scaffold’s microarchitecture, including average pore and window diameters, are crucial for cell-material interactions, degradation time, and mechanical properties of the scaffolds.^2−7^ Scaffolds with pore diameters smaller than required cause cell congestion, obstruction of mass transfer and neovascularisation. While increasing the pore diameter promotes cell motility and metabolic transport,^6^ scaffolds with larger or smaller pore sizes than required will result in a flattened cell shape by demonstrating a two-dimensional attachment behavior on the substrate (Figure 1), which may reduce cell adhesion and intracellular signal transmission.^8,9^ Furthermore, pore diameter also plays a crucial role in cell differentiation and gene expression.^10−12^ Pore size has been shown to be an effective factor in the differentiation of bone marrow stem cells into bone, chondrocytes, or smooth muscle cells, as well as adipose stem cells into chondrocytes or hepatic lines.^13^ As a consequence, having control over the morphological features of tissue engineering scaffolds and morphological characterization of the fabricated scaffolds is critically needed.

Electrospinning,^14,15^ 3D printing,^16^ solvent casting/particle leaching,^17,18^ phase separation,^19^ and freeze-drying^20^ have been widely used techniques for the fabrication of tissue engineering scaffolds. Alternatively, the emulsion templating technique has also gained increasing popularity in tissue engineering in recent years^21^ due to its various advantages, such as (i) providing up to 99% porosity,^22^ (ii) having a high degree of interconnectivity, (iii) enabling control of the morphological and mechanical characteristics of the scaffolds, and (iv) being suitable for combination with other scaffold fabrication techniques for the fabrication of structures with complex architectures.

**Figure 2:**
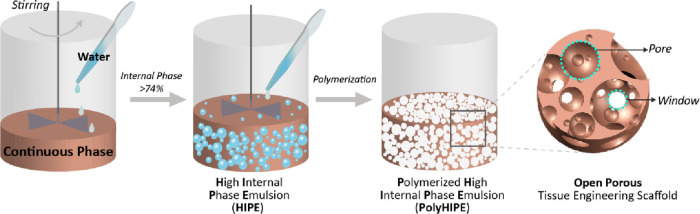
Fabrication steps of the polymerized high internal phase emulsion (PolyHIPE)-based scaffolds.

This method is based on creating a stable emulsion by mixing two immiscible liquids in the presence of a surfactant and solidifying the polymer phase (Figure 2). During solidification (or polymerization), the droplets in the interior phase of the emulsion serve as a pore template and are subsequently removed. The term “high internal phase emulsion (HIPE)” refers to an emulsion whose internal phase volume (total droplet volume) is more than 74%.^22^

Another crucial morphological feature, interconnectivity, is the term used to describe the degree of connection (window) of pores to neighboring pores of the scaffolds. Scaffolds are classified as open or closed porous scaffolds depending on the presence or absence of interconnects. Open pores are connected to both the surface and neighboring pores, whereas closed pores are not connected to either. From a biological point of view, open porosity is advantageous because it is essential for cell migration, nutrient transmission through the scaffold, waste removal, and the integration of the material with the host tissue.^7,23^ Polymerized HIPEs (also known as PolyHIPEs) are used to create open porous scaffolds (Figure 2) with an average pore diameter of 1–100 μm that permits cell infiltration.^24,25^

The morphological features of emulsion-templated scaffolds depend on various parameters related to the composition and processing conditions. For instance, parameters, including temperature, surfactant concentration,^26^ mixing speed, and polymer viscosity,^27^ directly impact the pore sizes of emulsion-templated scaffolds.^24^ Accordingly, by controlling each factor, scaffold morphology can be precisely engineered for specific biomedical applications.

The importance of scaffold morphology in terms of biological, mechanical, and biochemical aspects highlights the necessity of morphological characterization of the scaffolds. Various techniques are currently used for this purpose, such as scanning electron microscopy (SEM),^28^ flow and mercury porosimetry,^29^ gas pycnometry,^30^ nitrogen adsorption,^31^ and microcomputed tomography (micro-CT).^32^

One of the most widely used techniques for morphological characterization of scaffolds is SEM, which is a comparably accessible tool that allows the examination of more than one sample quickly and is relatively more cost-effective than methods such as micro-CT.^33^ However, the data obtained by SEM are qualitative; therefore, it is necessary to quantify these data to identify parameters such as average pore size, pore size distribution, average window diameter, window diameter distribution, and degree of interconnectivity and to compare these parameters for various experimental groups.^34,35^

In one of the most widely used techniques for quantifying SEM images, researchers randomly select a limited number of pores/windows on the SEM image of a scaffold^36−38^ and quantify the data using software such as ImageJ and FIJI to determine the average pore/window size.^7^ Although pore/window measurement via ImageJ is easy to apply in practice, it has significant drawbacks as the measurements are taken manually. In this application, measuring every individual pore/window in the SEM image is not practical, as it requires extensive time and effort. Therefore, determining a limited number of pores/windows for measurement and considering them representative of the whole sample is more widely preferred.^39,40^ Representative pores/windows are selected according to the user’s instructions to determine the average pore/window diameter, which may cause bias. Some studies may require the measurement of thousands of samples; ultimately, the rate of human error may increase in applications requiring a large volume of measurements.

Automated pore/window measurement systems from SEM images are needed to overcome these problems.^41^ Based on this need, studies on developing semi-automated systems have been reported. Using MATLAB, Jenkins et al. developed an algorithm called PoreScript that can measure pore sizes on the SEM images of scaffolds (fabricated by using salt leaching, gas foaming, or emulsion templating).^41^ PoreScript recognizes pores based on pixel intensity. It has been reported that PoreScript can measure the pore sizes of both open and closed cellular structures. This approach increases the number of samples that can be measured and reduces the user bias. However, there are certain drawbacks to this system. User input is required for parameters, such as pixel intensity threshold value and estimated pore size range. The accuracy of this algorithm decreases when there are considerable differences in the brightness of the pores. According to the results obtained with this algorithm, the difference between manual and semi-automated measurements was as high as 53%. Additionally, in this work, no study was conducted to determine the window diameter.^41^

Lo Re et al. developed a four-step algorithm that includes pre-processing, an auxiliary procedure for determining the threshold value, binarization and morphological analysis, and validation using MATLAB. The system was tested with particulate-leached polymeric scaffolds with different pore sizes and irregular pore morphologies. Only scaffolds with closed cellular morphologies were tested in that study. Additionally, it is a semi-automated system that requires pre-processing of the images.^42^

Deep learning, a technique based on artificial neural networks, has emerged as a powerful machine learning tool that enables computers to solve perceptual problems, such as visual objects and speech recognition. With the rapid data storage and parallelization features of deep learning, this technology’s ability to detect desired materials (images, sounds) and its recognition power contributed to its rapid adoption.^43,44^

**Figure 3:**
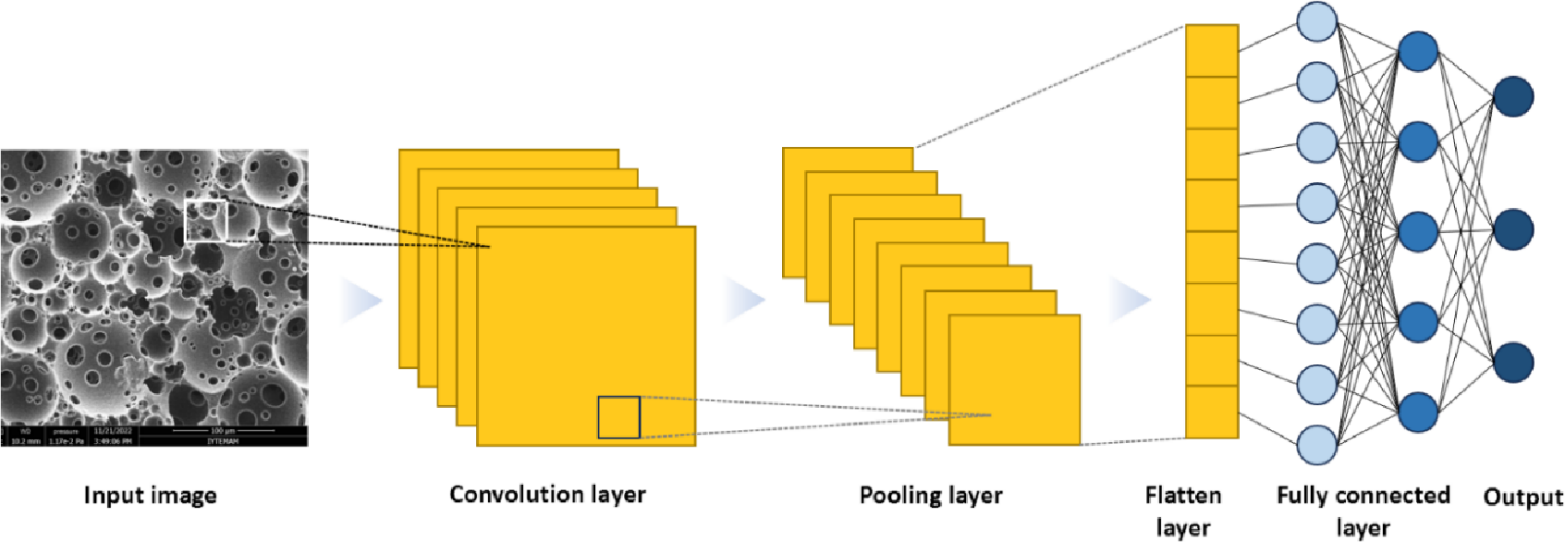
Schematic of convolutional neural networks.

Artificial neural networks, such as convolutional neural networks (CNNs), use multiple processing layers to recognize the structure of data sets (Figure 3). Each layer concentrates on and identifies a different concept, and as the number of hidden layers increases, the concepts learned also become abstracted.^44,45^ For instance, the pixels of an image are taken as inputs, and the edges might be defined in the first hidden layer by comparing them to the hues or luminosities of the nearby pixels. This determined information is then sent to the second hidden layer, and the second layer uses these data to find corners and contours. The information learned from this layer is transferred to the next hidden layer, and more specific details can be perceived so that the trained system can recognize the desired objects as a result of the learning process.^46^ Although CNN consists of a multilayer neural network, its structure is much more complex than that of traditional neural networks. It consists of additional convolutional, pooling, and flattening layers. A convolutional layer computes the similarity between small regions of the picture and a few learned kernels. The values of the close pixels are aggregated and merged into a single pixel in a pooling layer. As a result, the data becomes less complex, requiring less processing and leading to a feature selection that is resilient to even tiny changes.^47^ The flattening layer flattens the pooling layer’s final output, which is the transformation of a multidimensional input into a one-dimensional input.^48^ Then, those inputs are transferred to the fully connected neural network, which serves a role similar to that of the multilayer perceptron architecture.

Perera et al. used two regression CNNs to first distinguish between pores and particles and subsequently determine the pore size. Binary segmentation was used to detect the locations of the pores, and the YOLOv5 (you only look once) technique was used to quantify the pore diameters.^49^ Although highly accurate results were achieved with YOLOv5, only closed pores could be detected with this method. Thus, as in the other studies mentioned above, the window diameter cannot be measured automatically.^49^

**Figure 4:**
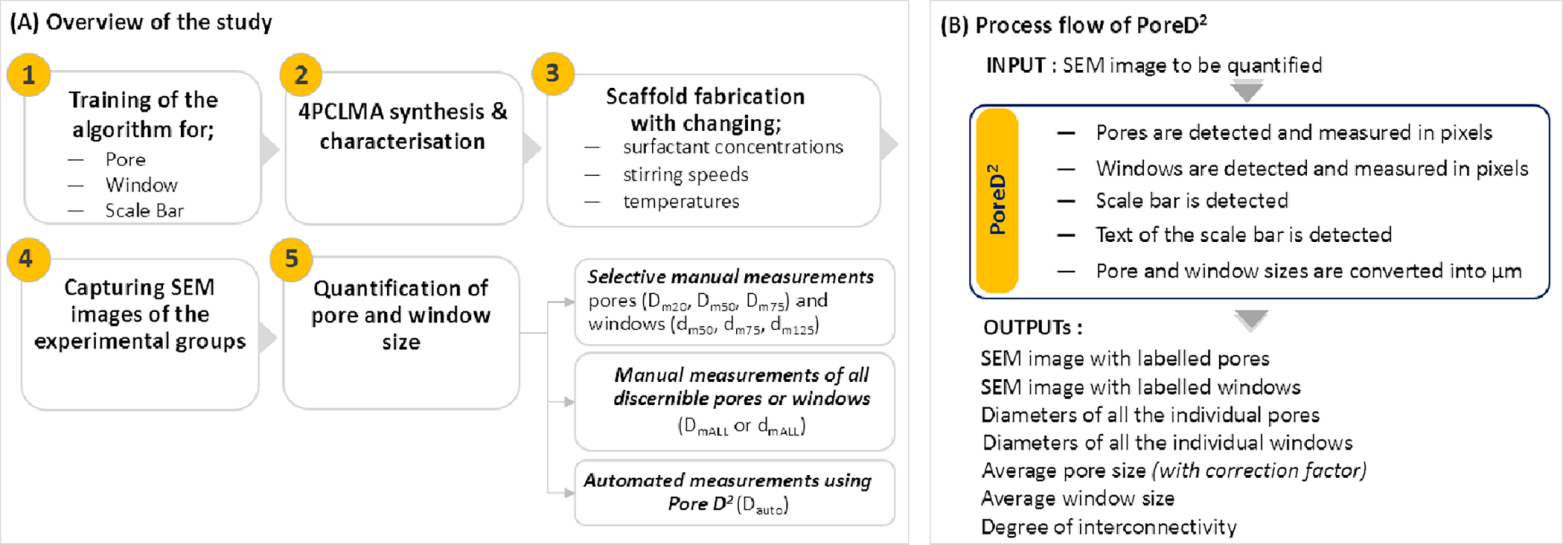
Schematic flowchart of (A) the study and (B) the process flow of Pore D^2^.

In the scope of this study, (i) First, a photocurable polymer was synthesized, and seven groups of PolyHIPEs were fabricated under different circumstances, such as by changing the surfactant concentration, stirring speed and temperature, to fabricate scaffolds with different morphologies. (ii) Then, four users quantified (blind quantification) 20, 50, and 75 pores and 50, 75, and 125 windows in the seven groups of PolyHIPEs to investigate the impact of the user and the number of counted pores and windows on the manually calculated average pore and window diameters. (iii) Afterward, we developed a completely automated system that performs quantitative analysis of the pore and window diameters of open porous emulsion templated scaffolds using deep learning techniques. For this purpose, the algorithm (Pore D^2^) was trained by introducing 3000 pores, 19800 windows, and 135 scale bars (tagged with online labeling tools) using the YOLOv5 model of the YOLO object recognition algorithm. In this study, the YOLOv5 model is chosen for the detection method since it provides great performance even in noisy environments.^50^ Moreover, its high inference speed^51,52^ makes YOLOv5 a desired approach for object detection applications. Additionally, EasyOCR is used for text recognition of scale bars. EasyOCR is a Python-based PyTorch library that uses a deep learning algorithm, resulting in great text recognition accuracy.^53^ (iv) Finally, all the discernible pores and windows in the SEM images of the seven experimental groups were measured both manually and with Pore D^2^. The effects of changing process parameters on scaffold morphology and the % deviation values between the automated and manual measurements were calculated and discussed (Figure 4).

## 2 Experimental Section

### 2.1 Materials

Pentaerythritol (98%), ε-caprolactone, tin(II) 2-ethyl hexanoate, triethylamine (TEA), methacrylic anhydride (MAAn), a photoinitiator (2,4,6-trimethyl benzoyl phosphine oxide/2-hydroxy-2-methylpropiophenone blend), and hydrochloric acid (HCl) were purchased from Sigma-Aldrich. 1,2-Dichloroethane (DCE) was purchased from Fisher Scientific (Pittsburgh, PA, USA). Polyglycerol polyricinoleate 4125 (PGPR) was kindly donated by Paalsgard (Juelsminde, Denmark).

### 2.2 Methods

#### 2.2.1 Training of the YOLO Object Detection Model for the Measurements of Pores, Windows, and Scale Bars

**Figure 5:**
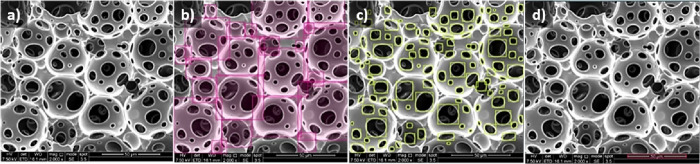
(a) Unlabeled and (b–d) pore, window, and scale bar labeled SEM images obtained using the online labeling tool (Roboflow).

The data sets were created with 50, 47, and 135 SEM images of previously prepared 4-arm polycaprolactone methacrylate (4PCLMA) based PolyHIPE samples for pore, window, and scale bar measurements, respectively. Before the training process, bounding boxes of pores, windows, and scale bars were produced by Roboflow (Roboflow, Inc., USA; Figure 5). EasyOCR is used for text recognition of scale bars. The data sets contain 3000 pores, 19800 windows, and 135 scale bars. The images were split into two parts, training and validation, at a ratio of 7:3. The training process was conducted using the Google Colaboratory (Colab) Python notebook, and YOLOv5 × 6 was used. The image size was 640, and we used a batch size of 16 for training. The default hyperparameters have been used.

#### 2.2.2 4PCLMA Synthesis

4PCLMA synthesis was conducted as explained in detail previously.^27,54,55^ Briefly, under nitrogen flow, pentaerythritol, and ε-caprolactone were added to a round-bottomed flask, and the mixture was heated to 160 °C using an oil bath while mixing. When the pentaerythritol was completely dissolved, the catalyst tin(II) 2-ethyl hexanoate was added, and the mixture was left overnight to form 4PCL before being removed from the oil bath and allowed to cool in the ambient atmosphere. 4PCL was dissolved in DCM, after which TEA was added. The reagents were stirred to ensure that all of the reagents were dissolved. The flask was placed in an ice bath. MAAn was dissolved in DCM and placed in a dropping funnel. When MAAn was completely dispensed, the ice bath was removed, and the mixture was maintained at room temperature (RT). The sample was then washed with an HCl solution and deionized water to remove the TEA, MAA, and salts that had formed. Almost all of the solvents were evaporated, three methanol washes were applied, and any remaining solvent was again removed using a rotary evaporator. 4PCLMA was stored in the freezer until further use.

#### 2.2.3 Preparation of Test Groups to Investigate the Effect of Surfactant Concentration, Stirring Speed, and Temperature on the Morphology of 4PCLMA PolyHIPEs

For the preparation of PolyHIPE, polymer (0.2 g), surfactant (10% PGPR w/w), solvent (dichloroethane, 150% w/w), and photoinitiator (10% w/w) were added to the glass vial; the mixture was stirred with a magnetic stirrer; and during the mixing step, water (1.5 mL) was added dropwise to create an emulsion. After mixing for 2 min, HIPE was poured into the molds, which were subsequently photocured with UV light.

Different sets of PolyHIPEs were prepared to evaluate the impact of temperature (RT, 37 °C, and 50 °C), mixing speed (350, 500, and 750 rpm), and surfactant concentration (5, 10, and 15%) on the pore and window diameter of the emulsion templated scaffolds (*while keeping the other parameters constant*). The conditions for the test groups are presented in Table 1.

**Table 1:** Sample Names and Preparation Conditions for the Test Groups to Investigate the Effect of Surfactant Concentration, Stirring Speed, and Temperature on the 4PCLMA PolyHIPE Morphology

sample name	surf. (%)	*T* (°C)	stirring (rpm)	*D*_mALL_ (μm)	*D*_auto_ (μm)	deviation^a^ (%)	*d*_mALL_ (μm)	*d*_auto_ (μm)	deviation^a^ (%)	DI (*d*_mALL_/*D*_mALL_)	DI (*d*_auto_/*D*_auto_)
I1	5^*^	RT^***^	350^**^	40.2	44.6	11	10.02	9.00	10	0.25	0.20
I2	10^*^	RT	350	32.3	35.3	9	7.72	8.66	12	0.24	0.25
I3	15^*^	RT	350	30.4	32.4	7	8.74	9.36	7	0.29	0.29
I4	5	RT	500^**^	29.9	34.3	14	7.25	7.35	1	0.24	0.21
I5	5	RT	750^**^	19.4	21.5	11	5.87	5.72	3	0.30	0.27
I6	5	37^***^	350	96.3	92.8	4	26.18	22.80	13	0.27	0.25
I7	5	50^***^	350	101.5	102.7	1	27.44	26.48	3	0.27	0.26

a Per cent deviations between *D*_mALL_ and *D*_auto_, *d*_mALL_ and *d*_auto_, respectively. (*D*_mALL_ and *d*_mALL_ are all the detectable pores and windows measured manually, respectively. *D*_auto_ and *d*_auto_ are all of the detectable pores and windows measured using Pore D^2^, respectively. DI: degree of interconnectivity. *: surfactant concentration, **: stirring speed, ***: process temperature comparison groups, respectively.)

#### 2.2.4 Scanning Electron Microscopy

SEM was used to investigate the microarchitecture of the scaffolds. The samples were cut by using a scalpel and placed on SEM pins with carbon pads. The samples were gold sputter-coated at 15 kV for 2.5 min to increase the conductivity. An FEI Inspect F SEM (Philips/FEI XL-20 SEM, Cambridge, UK) was used with 10 kV power.^56−58^

#### 2.2.5 Manual Measurement of the Pore and Window Diameters

Two different routes were followed for the manual measurements of the pores and windows. In the first route, we aimed to investigate how the quantified number of features and different users affect the quantification results. 20, 50, or 75 pores (*D*_m20_, *D*_m50_, and *D*_m75_, respectively) and 50, 75, or 125 windows (*d*_m50_, *d*_m75_, and *d*_m125_, respectively) were randomly selected from three different regions of each sample, and measurements were taken by four blind assessors using ImageJ. In the second part of the quantification process, all of the discernible pores/windows were manually measured to compare with the results of the algorithm (*D*_mALL_). Images at 1000× magnification were used where possible. A statistical correction factor (2/√3) was applied to the pore measurements to adjust for underestimation of diameter because of uneven sectioning,^59^ and average pore and window sizes were reported.

#### 2.2.6 Determining the Pore and Window Diameter Using a Deep Learning Technique (Pore D^2^)

After training the model (Section 2.2.1), the weights were saved as a ″.pt” file. The SEM images of the test groups were run through the model using the acquired weight, and the sizes of the discernible pores (D) and windows (d) were automatically detected, measured in pixels, and converted to μm using the algorithm Pore D^2^. During the fully automated measurements, both the height and width of the pores were measured, and in consideration of pores that did not fit the SEM frame, the height and width were compared, and the larger value was taken as the size of that pore. Moreover, the degree of interconnectivity (DI) was obtained from the ratio of average window diameter to average pore diameter (d/D).

## 3 Results and Discussion

### 3.1 Training of the YOLO Object Detection Model for Pore, Window, and Scale Bar Detection

**Figure 6:**
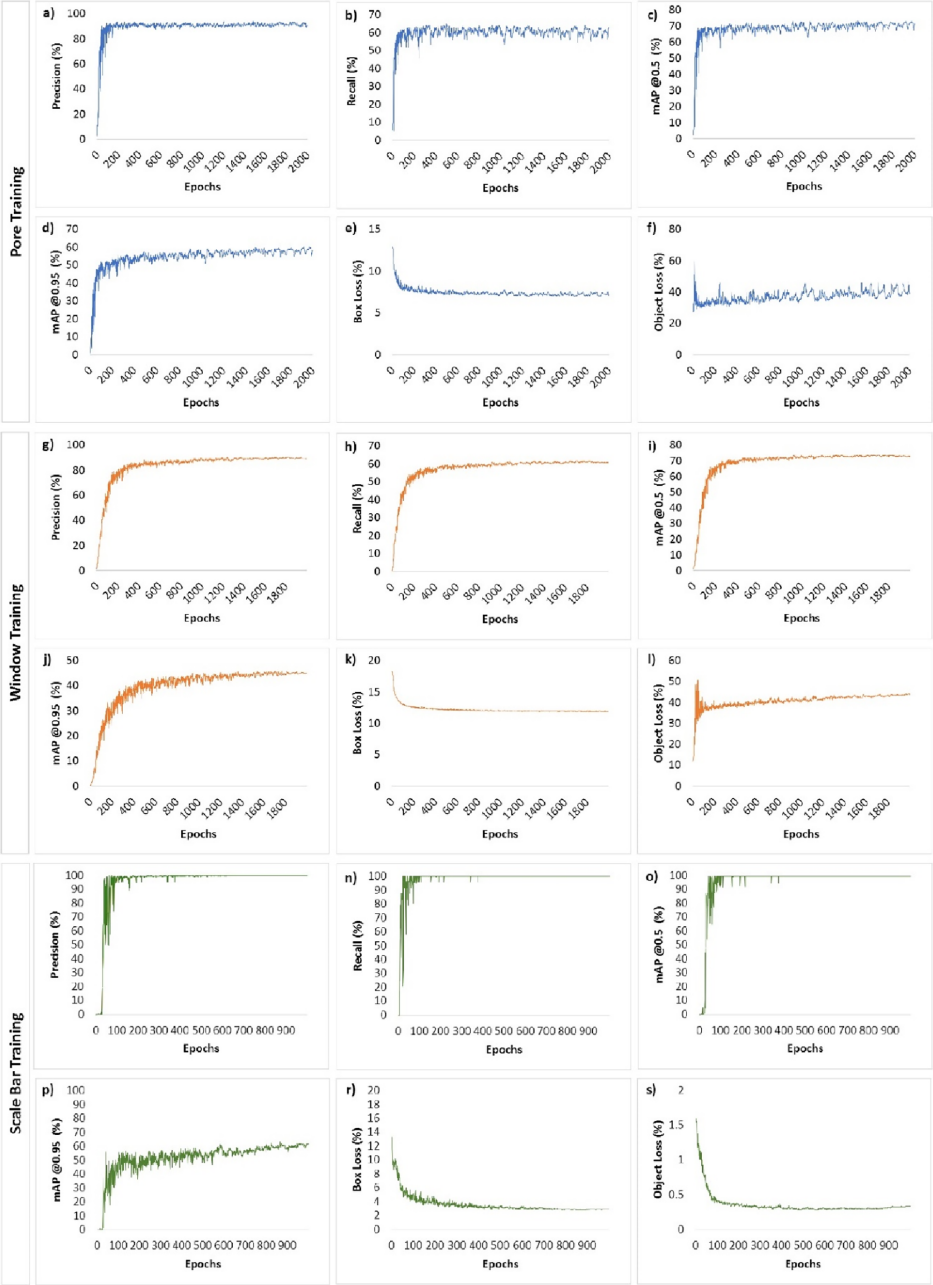
(a–f) Precision%, recall%, mAP @0.5 (%), mAP @0.95 (%), box loss (%), and object loss (%) results for pore training; (g–l) window training; and (m–s) scale bar training.

For the pore size measurements, the network was aimed to be trained for 5000 epochs; however, the training was terminated in the 2000 epochs, as no significant improvement was observed after the 1500 epochs. The class loss and mean average precision (mAP) at 50% of the intersection of union (IoU) were constantly monitored to evaluate the network’s performance. The IoU is calculated by comparing the detected box and the ground-truth box. On the other hand, for mAP@0.50 and mAP@0.95, the intersection of union overlap is 50 and 95%, respectively.^60^ The mAP (@50% of IoU) was 74.27% (Figure 6c), while the recall (Figure 6b) and precision (Figure 6a) were 61.75 and 91.91%, respectively. Moreover, the mAP (@95% of the IoU) reached 61.74% (Figure 6d). On the other hand, the box (Figure 6e) and object (Figure 6f) losses continuously decreased to 7.11 and 38.85%, respectively.

For the quantification of the windows, the precision (Figure 6g), the recall (Figure 6h), mAP (@50% of the IoU) (Figure 6i), and mAP (@95% of the IoU) (Figure 6j) were 90.34, 61.33, 73.52, and 45.54%, respectively. While the implementation of the 5000 epochs was also aimed at window training, the training was terminated in the 2000 epochs, because no significant improvement was observed after the 1500 epochs. Decreased box losses (Figure 6k) and object losses (Figure 6l) are also observed for window training: 11.90% of the losses are obtained from box loss, and 43.90% are obtained from object loss (Figure 6). When the results obtained from pore detection and window detection are compared, especially in mAP (@95% of the IoU), there is a significant decrease, which can be expected since window complexity is higher, and it is also observed that the system has difficulty detecting tiny windows. Those problems were also reflected in box and object loss, for which pore detection gave better results.

Similar results were obtained for scale bar training (Figure 6m–s). Although the number of trained labeled items for scale bar training was less than the number of labels for pore and window training when training compared to each other, scale bar training gave better results than others when mAP values (mAP @0.5 was 98% (Figure 6o) and mAP @0.95 was about 60% (Figure 6p)) were compared. Moreover, in scale bar detection, both precision% (Figure 6m) and recall% (Figure 6n) values are approximately 100%. It can be concluded that results were obtained with high accuracy. The reason behind this is likely that pore and window structures are more complex than other structures and the effects of contrast, hue, and shadows are greater on the training results.

### 3.2 Effect of Surfactant Concentration on PolyHIPE Morphology

The effects of changing process parameters, namely, surfactant concentration (5, 10, and 15%), stirring speed (350, 500, and 750 rpm), and temperature (RT, 35 °C, and 50 °C), on the morphology of the PolyHIPE scaffolds were studied. Seven different PolyHIPE compositions (Table 1) were developed and investigated using SEM.

**Figure 7:**
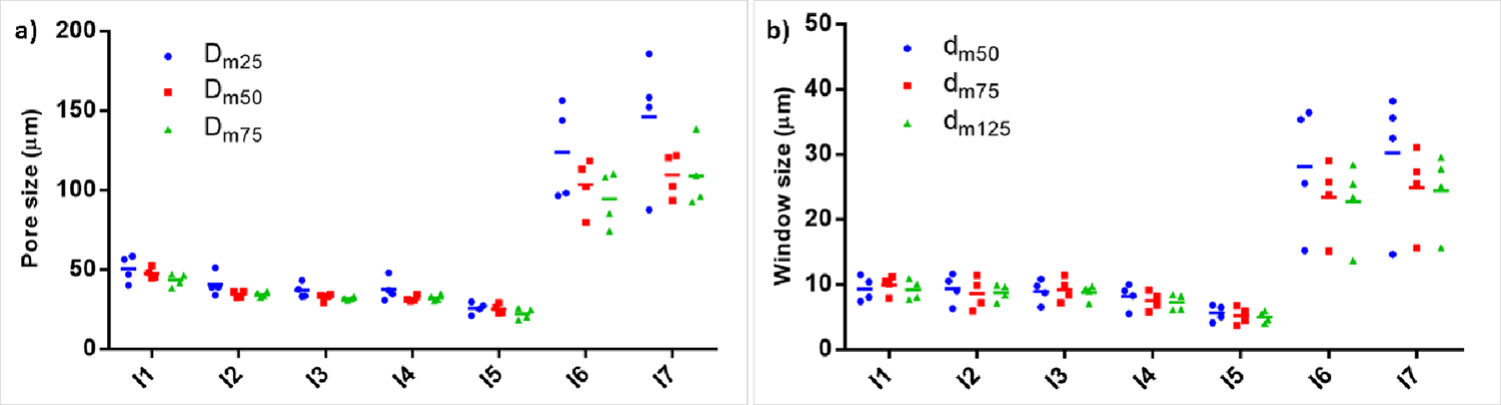
Manual blind measurements of the (a) pores and (b) windows by four diffent users. *D*_m20_: 20 pores, *D*_m50_: 50 pores, *D*_m75_: 75 pores measured manually. *d*_m50_: 50 windows, *d*_m75_: 75 windows, *d*_m125_: 125 windows measured manually.

First, we wanted to emphasize the importance and need for automated morphological quantification and tested the following hypothesis: the number of counted pores and different users (counting individuals) have an impact on the average calculated pore and window diameters. For this purpose, four users quantified (blind quantification) 20, 50, and 75 pores and 50, 75, or 125 windows of the seven groups of PolyHIPEs, and the quantification results were compared (Figure 7).

When PolyHIPEs were prepared using 5% surfactant (I1), the pore sizes were measured as 50.5, 47.6, and 43.4 μm when 20, 50, and 75 pores were measured manually, respectively. The measured average pore size decreased with an increasing number of counted pores. Indeed, according to our observations, if users are asked to count pores randomly, they tend to count larger pores, even though they try not to bias the pore selection. A similar trend was observed for the other groups and window measurements. Additionally, the data distribution shown in Figure 7 reveals that the impact of the individuals on the average measured pore and window diameter is significant. There is a significant difference in the quantified average pore and window diameters by different users, especially in I6 and I7. This is likely due to the larger pore size distributions in these groups.

**Figure 8:**
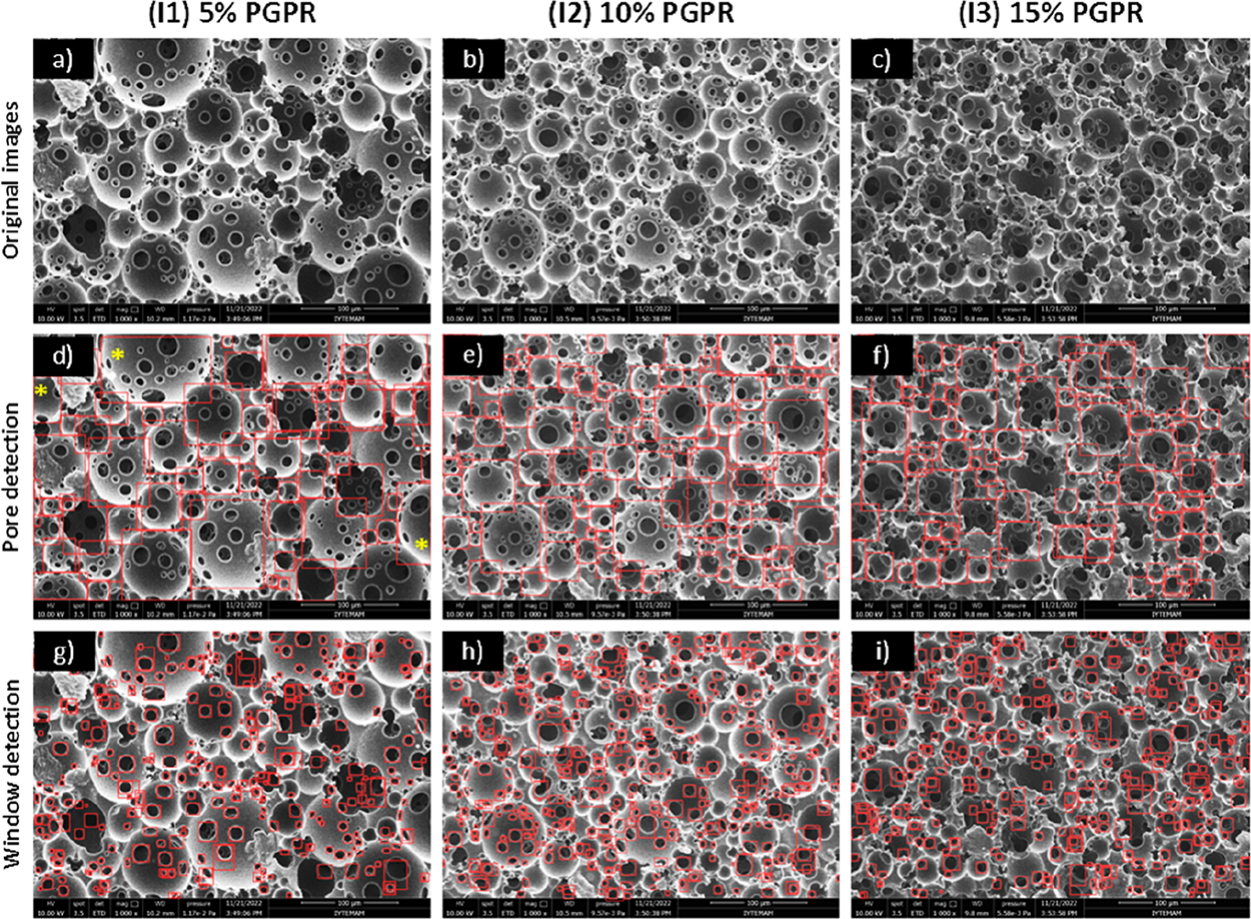
(a–c) SEM images showing the effect of surfactant concentration (other parameters were kept constant, RT, and mixing at 350 rpm) on PolyHIPE morphology, (d–f) pore, and (g–i) window detection results with the YOLO object detection algorithm. *: pores extending beyond the frame.

In the second part of the quantification process, we measured all the pores using our algorithm, Pore D^2^ (*D*_auto_), and compared them with *D*_mALL_, where we manually measured all of the discernible pores. Pore sizes were measured as 44.6, 35.3, and 32.4 μm in Pore D^2^ and 40.2, 32.3, and 30.4 μm manually for compositions prepared using 5, 10, and 15% PGPR (I1–I3), respectively (Figure 8).

**Figure 9:**
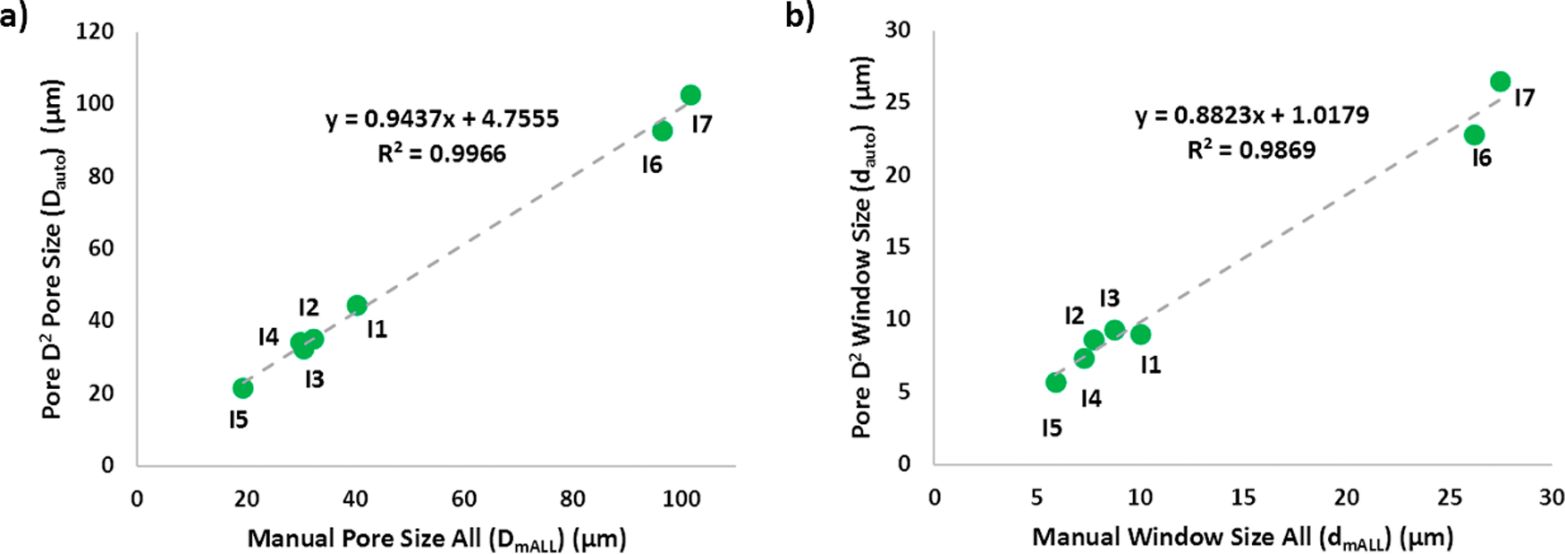
Average pore and window diameters obtained from automated (*D*_auto_, *d*_auto_) vs manual (*D*_mALL_, *d*_mALL_) measurements.

The percent deviation values between manual and automatic counting were 11, 9, and 7. When the *D*_mALL_ vs *D*_auto_ plot was drawn (Figure 9), the slope of the graph was calculated to be 0.9437, with an *R*^2^ value >0.99. If the manual and automated measurements fully align, the *R*-value and the slope would be 1. Thus, the results suggest a strong correlation between the two methods.

Following the training of the algorithm for pore detection, the code was initially written to label each pore and calculate the average of its width and height as the pore diameter. However, pores extending beyond the frame (in Figure 8d, labeled with a star) were observed to cause high errors. As PolyHIPEs generally give almost circular pores (the height-to-width ratio is approximately 1), in the second version, the code was written to label each pore, compare the height and width, and select the larger value as the pore diameter. In this way, we could calculate the pores extending beyond the frame in one direction more accurately.

When all of the quantified results were considered, a dramatic decrease in the pore size was observed for the scaffolds prepared with 5% surfactant concentration compared to the scaffold with 10% surfactant concentration; there was only a slight reduction when the surfactant concentration was increased to 15%. Increasing the surfactant concentration increases the stability of the HIPEs, which will cause a reduction in the average pore size of the PolyHIPEs.^26,40,61^

Finally, the average window sizes of the samples prepared with 5, 10, and 15% surfactant concentrations were measured as 10.02, 7.72, and 8.74 μm, respectively, via manual measurement and 9.00, 8.66, and 9.36 μm, with Pore D^2^. The % deviation values between manual and automatic counting were 10, 12, and 7%. When we compared the average window sizes of the groups, the surfactant concentration did not seem to have a significant effect on the window size. The slope and the *R*^2^ value for the *d*_mALL_ vs *d*_auto_ graph were calculated as ∼0.88 and ∼0.99, respectively, revealing a strong correlation between the manual and automated measurements.

### 3.3 Evaluation of the Effect of Stirring Speed on PolyHIPE Morphology

**Figure 10:**
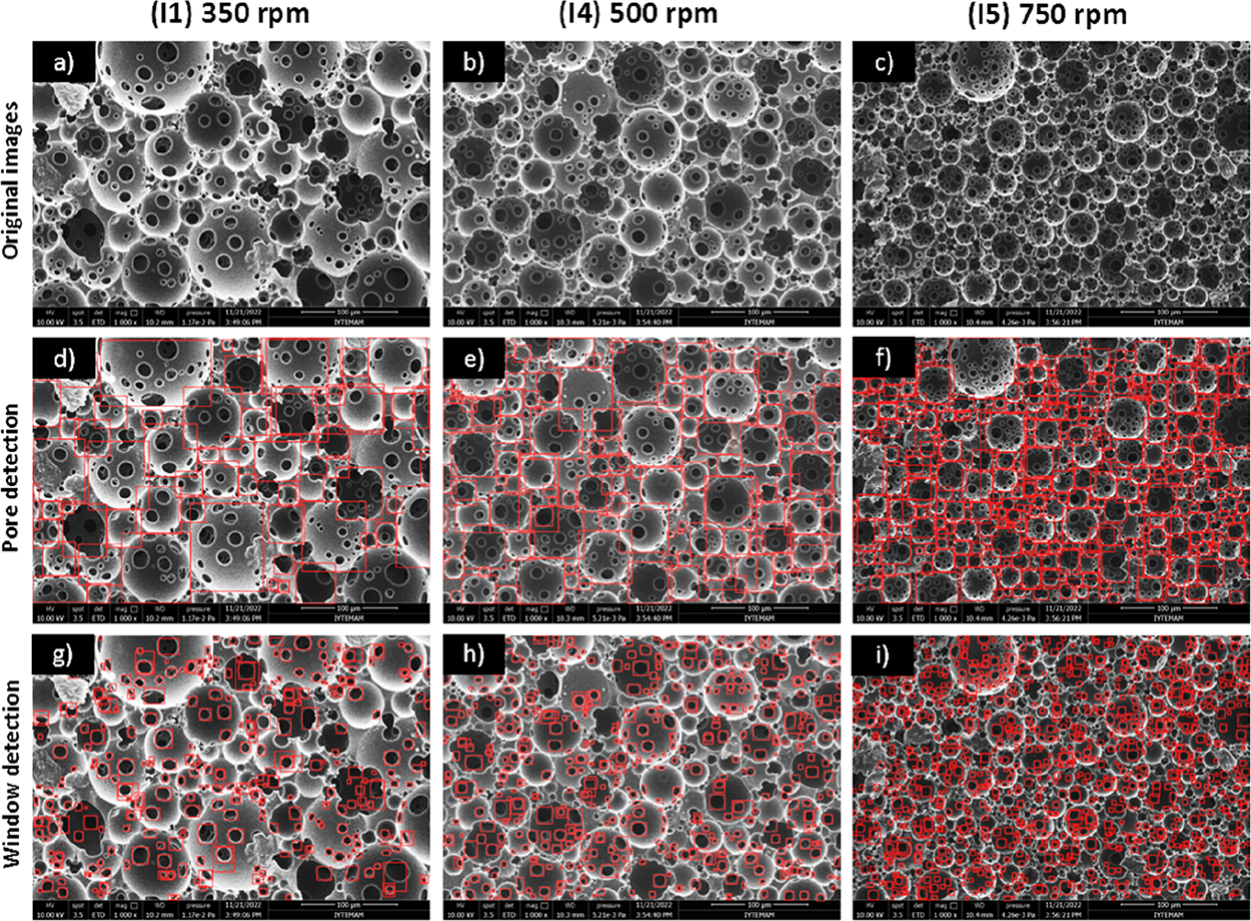
(a–c) SEM images showing the effect of stirring speed (other parameters were kept constant, RT and 5% PGPR) on the PolyHIPE morphology, (d–f) pore detection, and (g–i) window detection results with the YOLO object detection algorithm.

As shown in the SEM images of the 4PCLMA PolyHIPE scaffolds prepared using different stirring speeds (Figure 10), both the pore and window sizes decreased as the mixing speed increased (I1, I4, and I5). The average pore diameters of the PolyHIPEs prepared at 350, 500, and 750 rpm were 44.6, 34.3, and 21.5 μm with Pore D^2^ and 40.2, 29.9, and 19.4 μm, respectively, with automatic measurements. The percent deviation values between manual and automatic counting were 11, 14, and 11%. For the same group of samples, window sizes were measured as 9.00, 7.35, and 5.72 μm with Pore D^2^, and window sizes were measured manually as 10.02, 7.25, and 5.87 μm with % deviation values of 10, 1, and 3%, respectively.

An increase in mixing speed was anticipated to boost the shear stress applied to the emulsion and enhance droplet decomposition, which would lead to a decreased pore size distribution in the resultant PolyHIPE.^62,63^ A similar relationship between stirring speed and PolyHIPE pore size has also been reported in the literature for different materials.^24,40,61^

### 3.4 Evaluation of the Effect of Temperature on PolyHIPE Morphology

**Figure 11:**
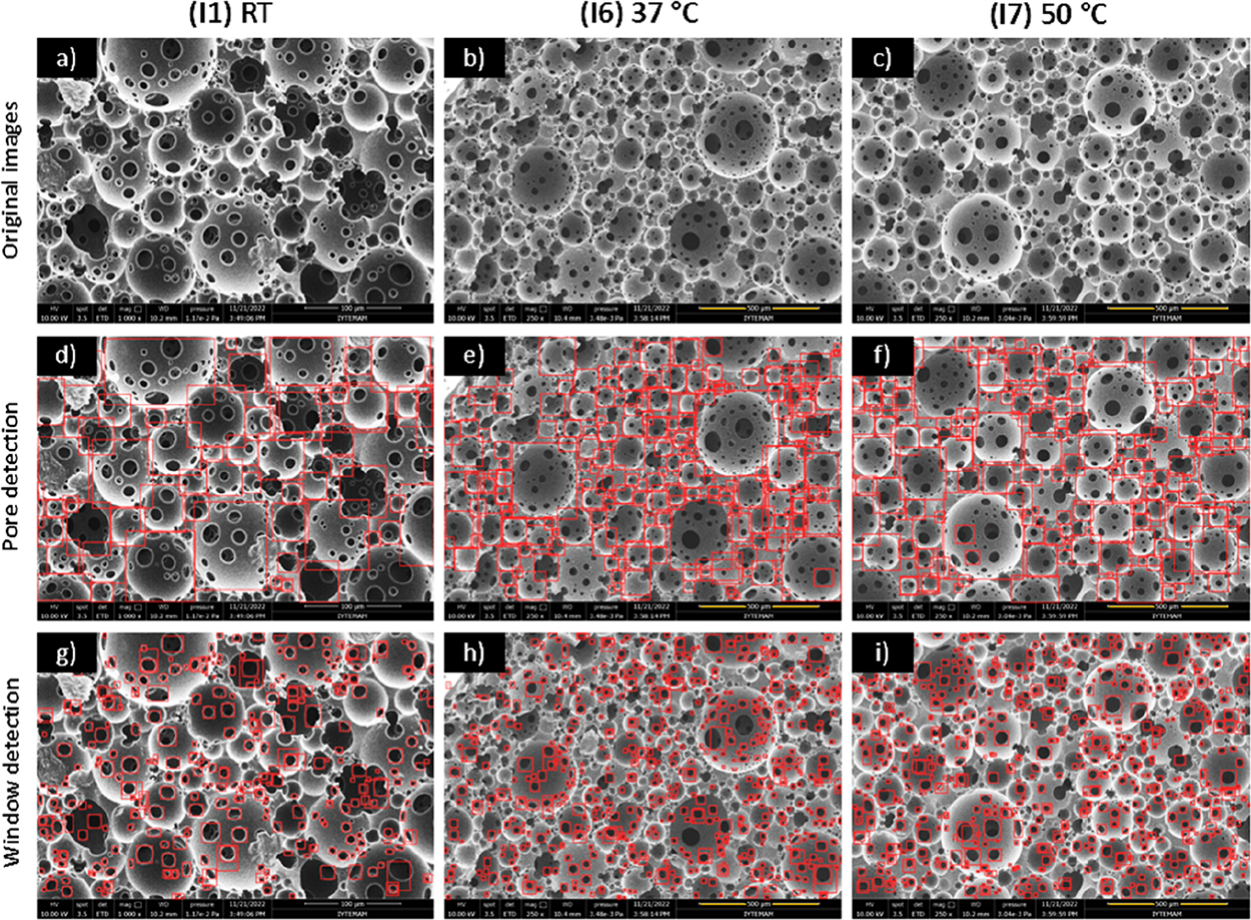
(a–c) SEM images showing the effect of temperature (other parameters were kept constant, 350 rpm and 5% PGPR) on the PolyHIPE morphology; (d–f) pore detection; and (g–i) window detection results with the YOLO object detection algorithm.

The morphologies of the PolyHIPEs prepared at different temperatures are shown in Figure 11a–11c (I1, I6, and I7). There was a significant difference in the pore sizes between the groups (please note that while the scale bars in the SEM images of the samples prepared at 37 and 50 °C are 500 μm, it is 100 μm for samples prepared at RT). The average pore diameters of the PolyHIPEs prepared at RT, 37 °C, and 50 °C were 44.6, 92.8, and 102.7 μm with Pore D^2^ and 40.2, 96.3, and 101.5 μm, respectively, with automatic measurements. Increasing the temperature causes a decrease in emulsion stability, which may cause an increase in the average pore size of PolyHIPEs.^24,63^

The average window sizes of the samples prepared at RT, 37 °C, and 50 °C were measured manually as 10.02, 26.18, 27.44 μm and 9.00, 22.80, 26.48 μm with Pore D^2^, respectively. The increase in window size with increasing temperature can be explained by the thermal agitation of water molecules, which increases contact and results in the merging of windows.^64^ Also, the velocity of the droplet (*v*) can be estimated according to Stoke’s equation (eq 1 where *D* is the droplet diameter under gravitational force, Δρ is the density difference between the water and oil phase, *n* is the viscosity of the oil phase, and *g* is the gravitational force)
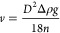
1

Increasing the temperature reduces the viscosity of the oil phase, which will eventually lead to an increase in the velocity of the water droplets, a reduction in the stability of HIPE and larger internal phase droplets.^65,66^

## 4 Conclusions

Morphological characterization of tissue engineering scaffolds is crucial for ensuring cell attachment, proliferation, and infiltration. In this study, we first showed that the number of pores and windows counted manually and the different users significantly affected the measured average pore and window diameters. To overcome this problem, a fully automated tool, Pore D^2^, was developed to quantify pores and window sizes of PolyHIPE scaffolds from SEM images using deep learning. Pore D^2^ allows us to obtain fast results with a high accuracy. Pore D^2^, an open-source software, is made publicly available (https://github.com/ilaydakaraca/PoreD2).

Overall, standard deviation values were not more than 14% in any group (1–14%) between manual and automated measurements of pores and windows. Despite all improvements in automated measurement, there are several misdirected, undetected, and multiple detected pores. On the other hand, manual measurements also involve human-sourced errors, as discussed in the introduction. As both methods have errors, it is not applicable to assume one way as a gold standard and compare the other with it. Here, we compared both methods to emphasize the similarity of the results, which are reported as % deviation.

The data sets consisting of 3000 pores and 19800 windows were labeled in the scope of this study. However, to increase the accuracy of the automated algorithm, the amount of data in the training data set can be increased. If the number of images in the data set is also increased, the algorithm can be better trained for images of different sizes, hue, and contrasts, and the accuracy would improve. The second option for increasing the accuracy is to modify the hyperparameters. In this study, default hyperparameters are used. As a result, mAP at 0.5 and mAP at 0.95 were found to be around 73–74 and 46–62%, respectively. Even though these values are within the range we initially aimed for, they can be improved for better performance, and modifying those hyperparameters decreases the possibility of overfitting, delays the occurrence of overfitting, and results in higher mAP scores. In addition to improving model performance, adjusting hyperparameters will reduce the number of misdirected, undetected, and multiple detected pores and windows, which will increase the model’s accuracy.

According to the results, increasing the mixing speed and surfactant concentration reduced the pore diameter, while increasing the temperature enhanced both the pore and window diameters. The morphology of emulsion templated scaffolds can be tuned by various parameters, and by using our fully automated algorithm, Pore D^2^, the time required for the morphological characterization of the scaffolds was greatly reduced, and highly accurate results were obtained. In future applications, the accuracy of these methods can be increased by introducing a higher number of training data, and the algorithm can also be trained for scaffolds fabricated with other scaffold fabrication routes.

## References

[ref1] Walles H.; Walles T. Extracellular Matrix as Biomimetic Biomaterial: Biological Matrices for Tissue Regeneration. Comprehensive Biomaterials 2011, 2, 361–367. 10.1016/B978-0-08-055294-1.00077-5.

[ref2] Murphy C. M.; Haugh M. G.; O’Brien F. J. The Effect of Mean Pore Size on Cell Attachment, Proliferation and Migration in Collagen-Glycosaminoglycan Scaffolds for Bone Tissue Engineering. Biomaterials 2010, 31 (3), 461–466. 10.1016/j.biomaterials.2009.09.063. 19819008

[ref3] Ruiz-Cantu L.; Gleadall A.; Faris C.; Segal J.; Shakesheff K.; Yang J. Characterisation of the Surface Structure of 3D Printed Scaffolds for Cell Infiltration and Surgical Suturing. Biofabrication 2016, 8 (1), 015016 10.1088/1758-5090/8/1/015016. 26930179

[ref4] Gupte M. J.; Swanson W. B.; Hu J.; Jin X.; Ma H.; Zhang Z.; Liu Z.; Feng K.; Feng G.; Xiao G.; Hatch N.; Mishina Y.; Ma P. X. Pore Size Directs Bone Marrow Stromal Cell Fate and Tissue Regeneration in Nanofibrous Macroporous Scaffolds by Mediating Vascularization. Acta Biomater 2018, 82, 1–11. 10.1016/j.actbio.2018.10.016. 30321630 PMC6258662

[ref5] Hosseinkhani M.; Mehrabani D.; Karimfar M. H.; Bakhtiyari S.; Manafi A.; Shirazi R. Tissue Engineered Scaffolds in Regenerative Medicine. World J. Plast Surg 2014, 3 (1), 3. 25489516 PMC4236978

[ref6] Klenke F. M.; Liu Y.; Yuan H.; Hunziker E. B.; Siebenrock K. A.; Hofstetter W. Impact of Pore Size on the Vascularization and Osseointegration of Ceramic Bone Substitutes in Vivo. J. Biomed Mater. Res. A 2008, 85 (3), 777–786. 10.1002/jbm.a.31559. 17896777

[ref7] Loh Q. L.; Choong C. Three-Dimensional Scaffolds for Tissue Engineering Applications: Role of Porosity and Pore Size. Tissue Eng. Part B Rev. 2013, 19 (6), 485–502. 10.1089/ten.teb.2012.0437. 23672709 PMC3826579

[ref8] Iordache F. Bioprinted Scaffolds. In Materials for Biomedical Engineering; Elsevier, 2019; pp 35–60. 10.1016/B978-0-12-816901-8.00002-X.

[ref9] Altuntaş E.; Özkan B.; Yener G. Porous Scaffolds. Nanobiomaterials Science, Development and Evaluation; Woodhead Publishing 2017, 27–59. 10.1016/B978-0-08-100963-5.00003-3.

[ref10] Matsiko A.; Gleeson J. P.; O'Brien F. J. Scaffold Mean Pore Size Influences Mesenchymal Stem Cell Chondrogenic Differentiation and Matrix Deposition. Tissue Eng. Part A 2015, 21, 486–4. 10.1089/ten.tea.2013.0545. 25203687

[ref11] Han Y.; Lian M.; Wu Q.; Qiao Z.; Sun B.; Dai K. Effect of Pore Size on Cell Behavior Using Melt Electrowritten Scaffolds. Front. Bioeng. Biotechnol. 2021, 9, 629270 10.3389/fbioe.2021.629270. 34277578 PMC8283809

[ref12] Reilly G. C.; Engler A. J. Intrinsic Extracellular Matrix Properties Regulate Stem Cell Differentiation. J. Biomech 2010, 43 (1), 55–62. 10.1016/j.jbiomech.2009.09.009. 19800626

[ref13] Oh S. H.; Kim T. H.; Im G. Il; Lee J. H. Investigation of Pore Size Effect on Chondrogenic Differentiation of Adipose Stem Cells Using a Pore Size Gradient Scaffold. Biomacromolecules 2010, 11 ( (8), ). 1948 10.1021/bm100199m. 20690707

[ref14] Jaganathan S. K.; Prasath Mani M.; Ayyar M.; Rathanasamy R. Biomimetic Electrospun Polyurethane Matrix Composites with Tailor Made Properties for Bone Tissue Engineering Scaffolds. Polym. Test 2019, 78, 105955 10.1016/j.polymertesting.2019.105955.

[ref15] Dikici S. A Sweet Way to Increase the Metabolic Activity and Migratory Response of Wound-Related Cells: Deoxy-Sugar Incorporated Natural Polymeric Fibres as a Potential Bioactive Wound Patch. Turk. J. Biol. 2021, 41 10.3906/biy-2108-27. 37533670 PMC10393099

[ref16] Jiao Z.; Luo B.; Xiang S.; Ma H.; Yu Y.; Yang W. 3D Printing of HA/PCL Composite Tissue Engineering Scaffolds. Advanced Industrial and Engineering Polymer Research 2019, 2 (4), 196–202. 10.1016/j.aiepr.2019.09.003.

[ref17] Park H. J.; Lee O. J.; Lee M. C.; Moon B. M.; Ju H. W.; Lee J. m.; Kim J. H.; Kim D. W.; Park C. H. Fabrication of 3D Porous Silk Scaffolds by Particulate (Salt/Sucrose) Leaching for Bone Tissue Reconstruction. Int. J. Biol. Macromol. 2015, 78 (2015), 215–223. 10.1016/j.ijbiomac.2015.03.064. 25849999

[ref18] Pashneh-Tala S.; Moorehead R.; Claeyssens F. Hybrid Manufacturing Strategies for Tissue Engineering Scaffolds Using Methacrylate Functionalised Poly(Glycerol Sebacate). J. Biomater Appl. 2020, 34 (8), 1114–1130. 10.1177/0885328219898385. 31930937

[ref19] Gay S.; Lefebvre G.; Bonnin M.; Nottelet B.; Boury F.; Gibaud A.; Calvignac B. PLA Scaffolds Production from Thermally Induced Phase Separation: Effect of Process Parameters and Development of an Environmentally Improved Route Assisted by Supercritical Carbon Dioxide. J. Supercrit. Fluids 2018, 136. 123 10.1016/j.supflu.2018.02.015.

[ref20] Zhang X.; Zhang Y.; Ma G.; Yang D.; Nie J. The Effect of the Prefrozen Process on Properties of a Chitosan/Hydroxyapatite/Poly(Methyl Methacrylate) Composite Prepared by Freeze Drying Method Used for Bone Tissue Engineering. RSC Adv. 2015, 5 (97), 79679–79686. 10.1039/C5RA14549J.

[ref21] (ALL = (emulsion templating)) AND ALL = (tissue engineering) – 204 – Web of Science Core Collection. https://www.webofscience.com/wos/woscc/summary/455c0e69-b08e-4d7a-be6a-f340e78cb5f0-73c5eab5/relevance/1 (accessed 2023–02–27).

[ref22] Richez A.; Deleuze H.; Vedrenne P.; Collier R. Preparation of Ultra-Low-Density Microcellular Materials. J. Appl. Polym. Sci. 2005, 96 (6), 2053–2063. 10.1002/app.21668.

[ref23] Ikada Y. Challenges in Tissue Engineering. J. R Soc. Interface 2006, 3 (10), 589 10.1098/rsif.2006.0124. 16971328 PMC1664655

[ref24] Aldemir Dikici B.; Claeyssens F. Basic Principles of Emulsion Templating and Its Use as an Emerging Manufacturing Method of Tissue Engineering Scaffolds. Front Bioeng Biotechnol 2020, 8, 875 10.3389/fbioe.2020.00875. 32903473 PMC7435020

[ref25] Aldemir Dikici B.; Chen M.-C.; Dikici S.; Chiu H.-C.; Claeyssens F. In Vivo Bone Regeneration Capacity of Multiscale Porous Polycaprolactone-Based High Internal Phase Emulsion (PolyHIPE) Scaffolds in a Rat Calvarial Defect Model. ACS Appl. Mater. Interfaces 2023, 15 (23), 27696–27705. 10.1021/acsami.3c04362. 37253168 PMC10273180

[ref26] Aldemir Dikici B.; Dikici S.; Claeyssens F. Synergistic Effect of Type and Concentration of Surfactant and Diluting Solvent on the Morphology of Emulsion Templated Matrices Developed as Tissue Engineering Scaffolds. React. Funct Polym. 2022, 180, 105387 10.1016/j.reactfunctpolym.2022.105387.

[ref27] Aldemir Dikici B.; Sherborne C.; Reilly G. C.; Claeyssens F. Emulsion Templated Scaffolds Manufactured from Photocurable Polycaprolactone. Polymer 2019, 175 (2019), 243–254. 10.1016/j.polymer.2019.05.023.

[ref28] Hassanajili S.; Karami-Pour A.; Oryan A.; Talaei-Khozani T. Preparation and Characterization of PLA/PCL/HA Composite Scaffolds Using Indirect 3D Printing for Bone Tissue Engineering. Materials Science and Engineering: C 2019, 104, 109960 10.1016/j.msec.2019.109960. 31500051

[ref29] Santos-Rosales V.; Gallo M.; Jaeger P.; Alvarez-Lorenzo C.; Gómez-Amoza J. L.; García-González C. A. New Insights in the Morphological Characterization and Modelling of Poly(ε-Caprolactone) Bone Scaffolds Obtained by Supercritical CO2 Foaming. J. Supercrit. Fluids 2020, 166, 105012 10.1016/j.supflu.2020.105012.

[ref30] Chachlioutaki K.; Karavasili C.; Adamoudi E.; Bouropoulos N.; Tzetzis D.; Bakopoulou A.; Fatouros D. G. Silk Sericin/PLGA Electrospun Scaffolds with Anti-Inflammatory Drug-Eluting Properties for Periodontal Tissue Engineering. Biomaterials Advances 2022, 133, 112723 10.1016/j.msec.2022.112723. 35474147

[ref31] Arabpour Z.; Baradaran-Rafii A.; Bakhshaiesh N. L.; Ai J.; Ebrahimi-Barough S.; Esmaeili Malekabadi H.; Nazeri N.; Vaez A.; Salehi M.; Sefat F.; Ostad S. N. Design and Characterization of Biodegradable Multi Layered Electrospun Nanofibers for Corneal Tissue Engineering Applications. J. Biomed Mater. Res. A 2019, 107 (10), 2340–2349. 10.1002/jbm.a.36742. 31161710

[ref32] Datta S.; Jana S.; Das A.; Chakraborty A.; Chowdhury A. R.; Datta P. Bioprinting of Radiopaque Constructs for Tissue Engineering and Understanding Degradation Behavior by Use of Micro-CT. Bioact Mater. 2020, 5 (3), 569–576. 10.1016/j.bioactmat.2020.04.015. 32373763 PMC7195521

[ref33] Haugen H. J.; Bertoldi S. Characterization of Morphology—3D and Porous Structure. Charact. Polym. Biomater. 2017, 21–53. 10.1016/B978-0-08-100737-2.00002-9.

[ref34] Ji Z. Use of Compositional and Combinatorial Nanomaterial Libraries for Biological Studies. Sci. Bull. (Beijing) 2016, 61 (10), 755–771. 10.1007/s11434-016-1069-z.

[ref35] Marquez A. L.; Gareis I. E.; Dias F. J.; Gerhard C.; Lezcano M. F. Methods to Characterize Electrospun Scaffold Morphology: A Critical Review. Polymers 2022, 14 (3), 467 10.3390/POLYM14030467. 35160457 PMC8839183

[ref36] Sherborne C.; Owen R.; Reilly G. C.; Claeyssens F. Light-Based Additive Manufacturing of PolyHIPEs: Controlling the Surface Porosity for 3D Cell Culture Applications. Mater. Des 2018, 156 (2018), 494–503. 10.1016/j.matdes.2018.06.061.

[ref37] Mert E. H.; Mert H. H. Preparation of PolyHIPE Nanocomposites: Revealing the Influence of Experimental Parameters with the Help of Experimental Design Approach. Polym. Compos 2021, 42 (2), 724–738. 10.1002/pc.25861.

[ref38] Durgut E.; Zhou M.; Dikici B. A.; Foudazi R.; Claeyssens F. Modifying Pickering Polymerized High Internal Phase Emulsion Morphology by Adjusting Particle Hydrophilicity. Colloids Surf. A Physicochem Eng. Asp 2024, 680, 132629 10.1016/j.colsurfa.2023.132629.

[ref39] Johnson D. W.; Langford C. R.; Didsbury M. P.; Lipp B.; Przyborski S. A.; Cameron N. R. Fully Biodegradable and Biocompatible Emulsion Templated Polymer Scaffolds by Thiol-Acrylate Polymerization of Polycaprolactone Macromonomers. Polym. Chem. 2015, 6 (41), 7256–7263. 10.1039/C5PY00721F.

[ref40] Moglia R. S.; Holm J. L.; Sears N. A.; Wilson C. J.; Harrison D. M.; Cosgriff-Hernandez E. Injectable PolyHIPEs as High-Porosity Bone Grafts. Biomacromolecules 2011. 12 3621 10.1021/bm2008839. 21861465 PMC3190649

[ref41] Jenkins D.; Salhadar K.; Ashby G.; Mishra A.; Cheshire J.; Beltran F.; Grunlan M.; Andrieux S.; Stubenrauch C.; Cosgriff-Hernandez E. PoreScript: Semi-Automated Pore Size Algorithm for Scaffold Characterization. Bioact Mater. 2022, 13, 1–8. 10.1016/j.bioactmat.2021.11.006. 35224287 PMC8843970

[ref42] Lo Re G.; Lopresti F.; Petrucci G.; Scaffaro R. A Facile Method to Determine Pore Size Distribution in Porous Scaffold by Using Image Processing. Micron 2015. 76 37 10.1016/j.micron.2015.05.001. 26026425

[ref43] Ravi D.; Wong C.; Deligianni F.; Berthelot M.; Andreu-Perez J.; Lo B.; Yang G. Z. Deep Learning for Health Informatics. IEEE J. Biomed Health Inform 2017, 21 (1), 4–21. 10.1109/JBHI.2016.2636665. 28055930

[ref44] Rusk N. Deep Learning. Nature Methods 2016 13:1 2016, 13 (1), 35–35. 10.1038/nmeth.3707.

[ref45] Lecun Y.; Bengio Y.; Hinton G. Deep Learning. Nature 2015 521:7553 2015, 521 (7553), 436–444. 10.1038/nature14539. 26017442

[ref46] Hao X.; Zhang G.; Ma S. Deep Learning. Int. J. Semant. Comput. 2016, 10 (3), 417–439. 10.1142/S1793351X16500045.

[ref47] Reimers C.; Requena-Mesa C. Deep Learning – an Opportunity and a Challenge for Geo- and Astrophysics. Knowledge Discovery in Big Data from Astronomy and Earth Observation: Astrogeoinformatics; Elsevier 2020, 251–265. 10.1016/B978-0-12-819154-5.00024-2.

[ref48] Melamane S.; Manadava T. T.; Manda A.; Luphade N.; Khamanga S. M. M.; Makoni P. A.; Demana P. H.; Matafwali S. K.; Witika B. A. Artificial Neural Network–Based Inference of Drug–Target Interactions. Nanotechnol. Principles Drug Target. Diagnosis 2023, 35–62. 10.1016/B978-0-323-91763-6.00015-1.

[ref49] Perera R.; Guzzetti D.; Agrawal V. Optimized and Autonomous Machine Learning Framework for Characterizing Pores, Particles, Grains and Grain Boundaries in Microstructural Images. Comput. Mater. Sci. 2021. 196 110524 10.1016/j.commatsci.2021.110524.

[ref50] *(2) (PDF)* A comparative study of YOLOv5 models performance for image localization and classification. https://www.researchgate.net/publication/363824867_A_comparative_study_of_YOLOv5_models_performance_for_image_localization_and_classification (accessed 2024–03–25).

[ref51] Fang Y.; Guo X.; Chen K.; Zhou Z.; Ye Q. Surface Knots on Sawn Timber. Bioresources 2021, 16 (3), 5390–5406. 10.15376/biores.16.3.5390-5406.

[ref52] Mahendrakar T.; Ekblad A.; Fischer N.; White R.; Wilde M.; Kish B.; Silver I. Performance Study of YOLOv5 and Faster R-CNN for Autonomous Navigation around Non-Cooperative Targets. IEEE Aerospace Conference Proceedings; IEEE 2022, 2022*-March*. 10.1109/AERO53065.2022.9843537.

[ref53] Vedhaviyassh D. R.; Sudhan R.; Saranya G.; Safa M.; Arun D. Comparative Analysis of EasyOCR and TesseractOCR for Automatic License Plate Recognition Using Deep Learning Algorithm. 6th International Conference on Electronics, Communication and Aerospace Technology, ICECA 2022 - Proceedings; IEEE, 2022, 966 971 10.1109/ICECA55336.2022.10009215.

[ref54] Aldemir Dikici B. Development of Emulsion Templated Matrices and Their Use in Tissue Engineering Applications; The University of Sheffield, PhD thesis, 2020. https://etheses.whiterose.ac.uk/27827/.

[ref55] Aldemir Dikici B.; Malayeri A.; Sherborne C.; Dikici S.; Paterson T.; Dew L.; Hatton P.; Ortega Asencio I.; MacNeil S.; Langford C.; Cameron N. R.; Claeyssens F. Thiolene- and Polycaprolactone Methacrylate-Based Polymerized High Internal Phase Emulsion (PolyHIPE) Scaffolds for Tissue Engineering. Biomacromolecules 2022, 23 (3), 720–730. 10.1021/acs.biomac.1c01129. 34730348

[ref56] Dikici S.; Aldemir Dikici B.; MacNeil S.; Claeyssens F. Decellularised Extracellular Matrix Decorated PCL PolyHIPE Scaffolds for Enhanced Cellular Activity. Integration and Angiogenesis. Biomater Sci. 2021, 9 (21), 7297–7310. 10.1039/D1BM01262B. 34617526 PMC8547328

[ref57] Cevik M.; Dikici S. Development of Tissue-Engineered Vascular Grafts from Decellularized Parsley Stems. Soft Matter 2024. 20 338 10.1039/D3SM01236K. 38088147

[ref58] Dikici S. Enhancing Wound Regeneration Potential of Fibroblasts Using Ascorbic Acid-Loaded Decellularized Baby Spinach Leaves. Polym. Bull. 2024, 1 10.1007/s00289-024-05185-1.

[ref59] Barbetta A.; Cameron N. R. Morphology and Surface Area of Emulsion-Derived (PolyHIPE) Solid Foams Prepared with Oil-Phase Soluble Porogenic Solvents: Span 80 as Surfactant. Macromolecules 2004, 37 (9), 3188–3201. 10.1021/ma0359436.

[ref60] Kolawole S.; Osakuade O.; Saxena N.; Olorisade B. K. Sign-to-Speech Model for Sign Language Understanding: A Case Study of Nigerian Sign Language. In IJCAI International Joint Conference on Artificial Intelligence; 2022. 10.24963/ijcai.2022/855.

[ref61] Dhavalikar P.; Shenoi J.; Salhadar K.; Chwatko M.; Rodriguez-Rivera G.; Cheshire J.; Foudazi R.; Cosgriff-Hernandez E. Engineering Toolbox for Systematic Design of Polyhipe Architecture. Polymers (Basel) 2021, 13 (9), 1479 10.3390/polym13091479. 34064400 PMC8124597

[ref62] Foudazi R. HIPEs to PolyHIPEs. React. Funct Polym. 2021, 164, 104917 10.1016/j.reactfunctpolym.2021.104917.

[ref63] Huš S.; Kolar M.; Krajnc P. Tailoring Morphological Features of Cross-Linked Emulsion-Templated Poly(Glycidyl Methacrylate). https://doi.org/10.1080/15685551.2015.1070503 2015, 18 ( (7), ), 698–703. 10.1080/15685551.2015.1070503.

[ref64] Carnachan R. J.; Bokhari M.; Przyborski S. A.; Cameron N. R. Tailoring the Morphology of Emulsion-Templated Porous Polymers. Soft Matter 2006, 2 (7), 608–616. 10.1039/b603211g. 32680240

[ref65] Bokhari M.; Carnachan R. J.; Przyborski S. A.; Cameron N. R. Emulsion-Templated Porous Polymers as Scaffolds for Three Dimensional Cell Culture: Effect of Synthesis Parameters on Scaffold Formation and Homogeneity. J. Mater. Chem. 2007, 17 (38), 4088–4094. 10.1039/b707499a.

[ref66] Paterson T. E.; Gigliobianco G.; Sherborne C.; Green N. H.; Dugan J. M.; MacNeil S.; Reilly G. C.; Claeyssens F. Porous Microspheres Support Mesenchymal Progenitor Cell Ingrowth and Stimulate Angiogenesis. APL Bioeng 2018, 2 (2), 26103 10.1063/1.5008556. PMC6481713 31069300

